# Deep-inspirational breath-hold (DIBH) technique in left-sided breast cancer: various aspects of clinical utility

**DOI:** 10.1186/s13014-021-01816-3

**Published:** 2021-05-13

**Authors:** Szilvia Gaál, Zsuzsanna Kahán, Viktor Paczona, Renáta Kószó, Rita Drencsényi, Judit Szabó, Ramóna Rónai, Tímea Antal, Bence Deák, Zoltán Varga

**Affiliations:** Department of Oncotherapy, University of Szeged, Korányi fasor 12, 6720 Szeged, Hungary

**Keywords:** Breast radiotherapy, Deep inspirational breath-hold (DIBH), Heart protection, Radiation lung damage, LAD protection

## Abstract

**Background:**

Studying the clinical utility of deep-inspirational breath-hold (DIBH) in left breast cancer radiotherapy (RT) was aimed at focusing on dosimetry and feasibility aspects.

**Methods:**

In this prospective trial all enrolled patients went through planning CT in supine position under both DIBH and free breathing (FB); in whole breast irradiation (WBI) cases prone CT was also taken. In 3-dimensional conformal radiotherapy (3DCRT) plans heart, left anterior descending coronary artery (LAD), ipsilateral lung and contralateral breast doses were analyzed. The acceptance of DIBH technique as reported by the patients and the staff was analyzed; post-RT side-effects including radiation lung changes (visual scores and lung density measurements) were collected.

**Results:**

Among 130 enrolled patients 26 were not suitable for the technique while in 16, heart or LAD dose constraints were not met in the DIBH plans. Among 54 and 34 patients receiving WBI and postmastectomy/nodal RT, respectively with DIBH, mean heart dose (MHD) was reduced to < 50%, the heart V_25 Gy_ to < 20%, the LAD mean dose to < 40% and the LAD maximum dose to about 50% as compared to that under FB; the magnitude of benefit was related to the relative increase of the ipsilateral lung volume at DIBH. Nevertheless, heart and LAD dose differences (DIBH *vs.* FB) individually varied. Among the WBI cases at least one heart/LAD dose parameter was more favorable in the prone or in the supine FB plan in 15 and 4 cases, respectively; differences were numerically small. All DIBH patients completed the RT, inter-fraction repositioning accuracy and radiation side-effects were similar to that of other breast RT techniques. Both the patients and radiographers were satisfied with the technique.

**Conclusions:**

DIBH is an excellent heart sparing technique in breast RT, but about one-third of the patients do not benefit from that otherwise laborious procedure or benefit less than from an alternative method.

*Trial registration*: retrospectively registered under ISRCTN14360721 (February 12, 2021)

**Supplementary information:**

The online version contains supplementary material available at 10.1186/s13014-021-01816-3.

## Background

Radiotherapy has played an essential role in the management of breast cancer for decades. Due to the availability of modern radiotherapy technologies, significant changes have occurred in radiotherapy practice focusing on optimized care on an individual basis [1, 2].

After breast-conserving surgery most of the patients need postoperative whole breast irradiation (WBI) with or without a tumor bed boost, while in low-risk cases partial breast irradiation (PBI) is sufficient. The need of chest wall (CW) irradiation is infrequent unless if combined with nodal irradiation, postmastectomy irradiation (PMI). The practice of nodal irradiation shows a broad spectrum from the sole irradiation of different axillary levels to that of all regional nodes depending on the risk status and surgery performed. Clinical studies indicate that while after sentinel lymph node biopsy (SNB) axillary block dissection may be replaced with axillary level I-II nodal radiotherapy in limited nodal involvement cases, full nodal radiotherapy although at a price of radiation sequelae, may contribute to improved survival of axillary lymph node positive cases [3, 4].

Most breast cancer patients become long-survivors, hence therapeutic interventions should not endanger the patients’ general health and well-being. The main radiation-related hazards are radiogenic heart and lung damage resulting in significant morbidity many years or decades after the radiotherapy [5–7]. The excess relative risk of secondary lung cancer or cardiac mortality has been estimated as 0.11 and 0.04 per one Gray increase of the dose to the whole lung and heart, respectively [8, 9]. Radiation-induced heart disease (RIHD) most frequently manifests in the damage of the coronary and capillary vessels of the heart which induces a progressive fibrotic process leading to circulatory changes with potentially fatal ischemic heart disease (IHD) [10]. The dose-dependent harmful effect of radiation exposure of the heart has been demonstrated in retrospective analyses and simulations of radiotherapies of breast cancer (BC) patients with IHD [7, 11]. The dose to the heart and hence RIHD incidence is higher in left-sided cases and the risk is more significant in young patients [12]. It is estimated that every 1 Gy mean heart dose increases the incidence of IHD event by 7.4% that may be potentiated by preexisting cardiovascular risk factors [6, 11] and smoking [8]. In a systematic review of contemporary publications, a radiation dose to the heart of 5.2 and 3.7 Gy in left and right sided cases, respectively, is still demonstrated while the dose to the ipsilateral lung is 9 Gy [8]. The risk of radiogenic heart damage is linearly dose-dependent, but no lower threshold with the absence of risk has been identified [1]. Hence all efforts should be made to avoid or lessen heart exposure as much as possible.

There are many approaches to protect the heart from radiation exposure. Prone positioning and the breath-holding techniques operate by separating the heart and the radiation fields; the advanced IMRT and proton irradiation techniques are not widely applied, while the reduction of the volume to be irradiated during partial breast irradiation (PBI) or the omittance of radiotherapy are options in low-risk cases. These methods result in variable effects on lung and heart exposures: while prone radiotherapy dramatically reduces lung doses, heart doses individually differ [13–15].

The breath-holding technique first described in breast cancer in 2001 [16] only recently became widespread [17]. Its greatest impact is reduced dose to the heart and LAD, and to a lesser extent to the lung [17, 18]. Nevertheless, the magnitude of benefit individually varies according to the patient’s anatomical features and lung capacity [18]; occasionally cases with the absence of advantage [16] or even elevated heart doses [19] have been described. Since earlier we found prone positioning individually helpful for heart sparing in the RT of left breast cancer cases (13–15), we wished to study the practical aspects of DIBH to find out its optimal place in routine practice. We tested the feasibility of the method among all patients, analyzed the relative dosimetry benefits according to the indications, and in the WBI only cohort, analyzed nodal coverage; also, radiation lung changes were prospectively followed.

## Methods

This prospective cohort study had been approved by the Institutional Ethics Review Board of the University of Szeged (#272/2017), and all the enrolled patients gave their written informed consent to participation. Inclusion criteria were left-sided breast cancer needing postoperative WBI/PMI and informed consent, while the exclusion criteria were the presence of chronic obstructive pulmonary disease, bronchial asthma or other severe comorbidity that would hinder cooperation during DIBH (extreme obesity, mental disorder, hypacusis).

For DIBH, the protocol of the voluntary breath-holding technique as described by Bartlett et al. was followed [20; http://www.jove.com/video/51578/]. All enrolled patients were assessed for baseline breath-holding capacity and trained for DIBH by a physiotherapist (JS), who reinvited the patient for further support if needed. Those patients who could not practise breath-holding for a minimum of 20 s were withdrawn from DIBH. These, together with those who despite sufficient breath-hold did not have improved heart and/or LAD doses (and hence DIBH was irrelevant) were excluded from further analyses.

Planning CT series were acquired in supine position with the arms elevated under both normal breathing and DIBH; in WBI cases CT was performed also in prone position. In-room lasers, skin marks, and verbal instructions through an audiovisual system with high performance video cameras (RMC-190 controller, 2 PTC-120 robotic HD cameras, Datavideo Technologies Co., Taiwan) ensured consistent breath-hold; beam-gating was applied manually.

Target volumes and organs at risk (OARs) were outlined in all CT series, and treatment plans were generated in all setups. In the first series of 37 cases irradiated with DIBH technique, portal imaging 3-times a week with 2 orthogonal setup beams of kilovoltage photon energy was performed, necessary couch translations were recorded and inter-fractional systematic and random setup errors were calculated; also, daily treatment times were recorded, and questionnaires regarding the comfort of the RT procedure (patients, RT fractions 6 and 25, cumulative score 0–9) and the efforts needed from the staff during planning CT or RT (radiographers, planning CT and RT fractions 6 and 25, cumulative score 0–12) similarly to Bartlett et al. were completed [20]. High scores indicated satisfactory acceptance (Additional file 1).

### Radiotherapy and dosimetry data

**Fig. 1:**
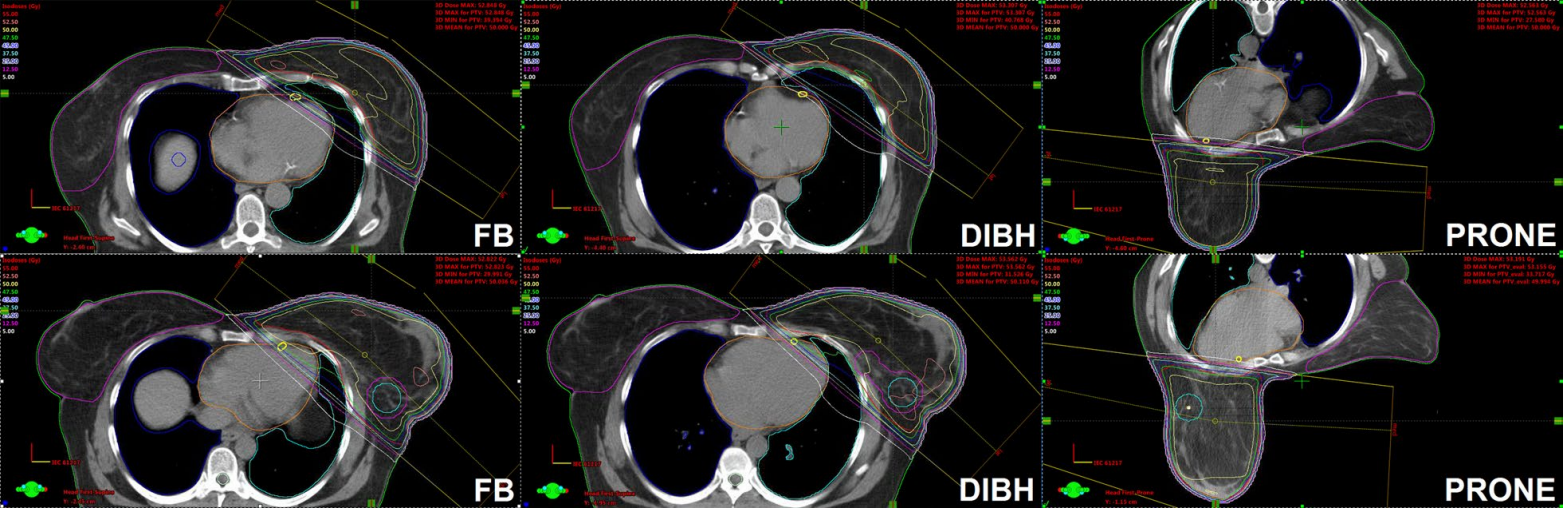
Three-dimensional conformal whole breast irradiation plans of 2 cases illustrating the benefit (upper series) or the lack of benefit (lower series) of the DIBH manoeuvre; transversal slices are shown during free-breathing (FB), DIBH and in the prone position (PTV contoured in red, heart contoured in orange, LAD contoured in yellow)

Radiotherapy techniques and facilities were described previously [13–15]. Briefly, patients were positioned on the supine thorax and prone breast modules (WBI cases) of the AIO (All In One) Solution (ORFIT, Wijnegem, Belgium) system without mask fixation. Planning CT images were acquired throughout the entire planning volume. Target volumes (whole breast or chest wall with or without nodal regions including axillary levels I-IV and internal mammary lymph nodes) and OARs i.e. the heart, LAD, ipsilateral lung, contralateral breast were contoured according to the European Society for Radiotherapy and Oncology (ESTRO) guidelines [21]. The aim was to achieve equivalent target and nodal volume contouring among all setups. The main objectives were a mean dose to the planning target volume (PTV) of 50 Gy (25 fractions), and V_47,5 Gy_ ≥ 90%, V_53,5 Gy_ ≤ 1%, while the dose constraints were the following: heart mean dose < 2.5 Gy, heartV_25Gy_ < 3%, LAD mean dose: < 12 Gy, lung mean dose < 10 Gy (WBI) and < 16 Gy (nodal RT), lung V_20 Gy_ < 15% (WBI) and 30% (nodal RT), contralateral breast V_10Gy_ < 5%. All plans were generated in the Varian Eclipse v13.6 (Varian Oncology Systems, Palo Alto, CA, USA) treatment planning system with the Analytical Anisotropic Algorithm (AAA) v8.0. 3DCRT plans applyed opposing tangential 6MV photon fields set up isocentrically and a median of 2 (1–3) 6/10 MV segmental fields (WBI, CW) (Fig. 1). For nodal irradiation, a single matched field was used. Sequential tumor bed boosts given according to the protocol were not included in the dosimetric comparisons. Radiotherapy was delivered with a Varian TrueBeamSTx (Varian Oncology Systems, Palo Alto, CA, USA) linear accelerator.

For plan evaluation, conformity and homogeneity indices [14, 22] were calculated as follows: CN = $\frac{{TV_{RI} }}{TV} \times \frac{{TV_{RI} }}{{V_{RI} }}$(TV: Target volume, i.e. PTV; TV_RI_: Target volume covered by the reference isodose; V_RI_: Volume of the reference isodose, ideal is 1),

Homogeneity Index (HI) [16] (D_2%_, D_50%_, D_98%_ = dose received by 2%, 50% and 98% of PTV, respectively, ideal is 0):

$$HI = \frac{{D_{2\% } - D_{98\% } }}{{D_{50\% } }}$$

The following dose-volume parameters of the PTV and OARs were collected: heart mean dose, heart V_25 Gy_, LAD mean dose, LAD maximum dose, ipsilateral mean lung dose, ipsilateral lung V_20 Gy_, contralateral breast V_10 Gy_. In further analyses we compared heart and LAD dose reductions (the differences between the heart mean dose, heart V_25 Gy_ and LAD mean dose in RT plans under FB *vs.* DIBH, respectively) with the relative increase of the ipsilateral lung volume.

In the WBI only cohort, nodal regions were retrospectively contoured both in the DIBH and free breathing (FB) planning CTs for nodal dosimetry (V_25 Gy_, V_45 Gy_, V_47.5 Gy_) comparisons [21, 23].

### Follow-up of patients including lung density change measurements

Data on clinical symptoms, skin changes and breast fibrosis were collected in the first cohort of patients at the completion of RT, and 3 months and 1 year thereafter using the CTCAE v. 4 system. Also, chest CTs under FB were performed at these time points for assessing post-irradiation lung changes as described [24]. Briefly, CT scans were evaluated both visually (category 0: no visible changes, category 1: increased density, hazy opacity, category 2: strand-like thickening) and with measuring the density of the lungs in a CT slice at the level of the left heart ventricle in the area outlined by the chest wall and a line between the edge of the sternum and the midheight of the chest in comparison with the planning CT. Density measurements were performed by excluding visible changes, and were corrected by subtracting the density of the unexposed contralateral lung. Visual assessment scores and mean lung density changes (MLDC) were analyzed and compared to the same parameters in a previous cohort of patients irradiated between 2010 and 2011 with 3DCRT under FB in supine position.

### Statistical analysis

Patient-related and dosimetry data were summarized using descriptive statistics; quantitative variables were expressed as mean ± SD. For the comparison of data, repeated ANOVA test (3 variables) and paired t-test (2 variables) were used. Lung density changes were analyzed with one-way ANOVA, while visual scores in the DIBH *vs.* FB groups with the chi-square test. The effects of patient-related variables or RT categories on DIBH-related dosimetry benefit were evaluated with linear regression.

## Results

According to the inclusion and exclusion criteria, 130 patients were enrolled between January 2018 and November 2019, nevertheless, among them 42 patients did not partake in DIBH and hence were not included in the dosimetry analysis of DIBH cases. Twenty-six patients were not suitable for DIBH (18 could not withhold breathing for 20 s, 3 patients were stressed, 2 withdrew consent, 1 could not follow the instructions, 1 was diagnosed with pulmonary embolism following enrollment and 1 with disease progression), while in 16 cases OAR doses were apparently not acceptable despite the DIBH manoeuvre. Out of the 16 patients with suboptimal 3DCRT plans under DIBH, 10 received RT with an alternative technique in supine position, and 6 received 3DCRT in prone position. Altogether 88 patients received RT under DIBH, their age was 57.3 ± 11.7 years (mean ± SD), their weight, length and BMI were 72.5 ± 13.6 kg, 163.6 ± 7.1 cm and 27.1 ± 5.1 kg/m^2^ (mean ± SD), respectively. Among them 35 received chemotherapy before the RT, and 56 received endocrine therapy during (aromatase inhibitors) or after (tamoxifen) the RT.

### Dosimetry data

In the group of 88 patients who received 3DCRT under DIBH, MHD was reduced by > 50%, the heart V_25 Gy_ by > 80%, the LAD mean dose by > 60% and the LAD maximum dose by about 50% as compared to that under FB (Table 1). The extent of dose reduction did not differ according to the indication of WBI, PMI or nodal RT. Lung doses were lower under DIBH than under FB and were dramatically reduced in the prone RT plans. The dose to the contralateral breast did not significantly change according to DIBH *vs.* FB. Nevertheless, heart and LAD dose differences (DIBH *vs.* FB) individually varied. Among the WBI cases at least one dose parameter was more favorable in the prone plan or in the supine WBI + FB plan in 15 or 4 cases, respectively; differences were numerically small (Table 2/A). None of the heart or LAD doses were superior under FB in the group of patients receiving WBI/CW + nodal RT. As mentioned earlier, in altogether 16 cases of all enrolled patients dose constraints were not met in the supine 3DCRT + DIBH plans; these patients received irradiation using an alternative technique with improved dose parameters as listed in Table 2/B.

**Table 1:** Dosimetry data among patients treated with 3DCRT and DIBH

n = 88	WBI (n = 54)							WBI + nodal/PM RT (n = 34)		
	FB (1)	DIBH (2)	Prone (3)	*p*	*p* (post hoc tests)			FB	DIBH	*p*
					1 vs 2	1 vs 3	2 vs 3			
PTV HI	0.102 ± 0.017	0.101 ± 0.012	0.105 ± 0.012	0.214	0.362	0.381	0.067	0.122 ± 0.011	0.129 ± 0.013	0.215
Heart mean dose (Gy)	3.16 ± 1.58	1.38 ± 0.37	2.02 ± 1.15	< 0.001	< 0.001	< 0.001	< 0.001	4.87 ± 1.21	2.13 ± 0.57	< 0.001
Heart V25 Gy (%)	4.13 ± 3.13	0.46 ± 0.51	1.44 ± 2.21	< 0.001	< 0.001	< 0.001	0.009	6.42 ± 2.43	1.23 ± 0.91	< 0.001
LAD mean dose (Gy)	13.05 ± 8.30	4.55 ± 1.95	9.98 ± 7.16	< 0.001	< 0.001	0.098	< 0.001	18.95 ± 7.03	7.47 ± 4.39	< 0.001
LAD max dose (Gy)	39.81 ± 14.52	18.92 ± 12.86	31.99 ± 14.15	< 0.001	< 0.001	0.021	< 0.001	47.03 ± 7.56	31.86 ± 10.83	< 0.001
Ipsilateral lung mean dose (Gy)	7.01 ± 1.97	6.37 ± 1.44	0.90 ± 0.63	< 0.001	< 0.001	< 0.001	< 0.001	14.29 ± 2.32	11.29 ± 1.91	< 0.001
Ipsilateral lung V20Gy (%)	12.11 ± 4.31	10.65 ± 3.17	0.65 ± 1.14	< 0.001	< 0.001	< 0.001	< 0.001	28.66 ± 5.36	21.64 ± 4.64	< 0.001
Contralateral breast mean dose (Gy)	0.36 ± 0.34	0.46 ± 0.50	0.59 ± 0.54	0.084	0.098	0.033	0.261	1.45 ± 1.31	1.4 ± 1.07	0.755
Contralateral breast V10 Gy (%)	0.46 ± 0.99	0.82 ± 1.59	0.76 ± 1.31	0.435	0.172	0.287	0.873	2.89 ± 3.32	3.07 ± 3.38	0.685

**Table 2:** Heart and LAD doses in patients for whom DIBH did not provide advantage

n = 88	WBI (n = 54)				WBI/CW + nodal (n = 34)	
	FB equal/better than DIBH		Prone equal/better than DIBH		FB equal/better than DIBH	
	mean difference (range)	n	mean difference (range)	n	mean difference (range)	n
Mean heart dose (Gy)	0.63 (0.63–0.63)	1	0.36 (0.00–0.89)	7	–	0
Heart V25Gy (%)	0.59 (0.00–1.19)	2	0.44 (0.00–1.88)	15	–	0
LAD mean dose (Gy)	0.53 (0.14–1.62)	5	1.91 (0.00–5.10)	7	–	0
LAD max dose (Gy)	6.18 (3.61–8.76)	2	8.16 (0.11–16.15)	6	–	0

Patient #	Target volume	Technique	Heart mean dose (Gy)			Heart V25Gy (%)			LAD mean dose (Gy)			LAD max dose (Gy)		
			DIBH	FB	Prone	DIBH	FB	Prone	DIBH	FB	Prone	DIBH	FB	Prone
1	WB	IMRT	2.75	6.99	3.87	2.94	11.79	5.02	14.99	22.84	19.73	49.96	50.45	48.66
2	WB	Prone	2.06	4.68	0.97	2.15	3.45	0	10.99	12.42	3.8	32.23	48.09	8.52
3	WB	Prone	2.96	2.98	1.66	2.63	2.55	0.02	18.84	20.53	3.75	46.73	45.83	22.19
4	WB	Prone	5.54	5.98	1.19	8.25	9.21	0	20.73	19.66	3.45	48.73	48.53	11.64
5	WB	Prone	3.42	5.16	1.39	4.92	8.79	0.03	24.23	37.43	3.52	49.11	50.37	9.66
6	WB	Prone	3.33	4.57	1.06	5.44	7.63	0	22.42	25.18	3.45	48.28	48.31	6.76
7	WB	Prone	5.34	5.43	1.39	10.37	10.05	0.28	21.55	19.29	5.83	50.7	49.97	31.57
8	WB/CW + nodal	IMRT	3.58	3.91		4.09	4.88		30.87	28.64		46.69	46.27	
9	WB/CW + nodal	IMRT	4.18	7.56		5.11	12.27		21.66	26.59		46.71	49.06	
10	WB/CW + nodal	IMRT	6.55	8		9.32	12.57		38.07	40.37		48.61	47.07	
11	WB/CW + nodal	IMRT	5.59	9.46		7.78	16.49		23.94	21.46		48.11	48.95	
12	WB/CW + nodal	3DCRT	7.93	6.81		13.51	11.67		15.32	17.16		50.63	49.71	
13	WB/CW + nodal	3DCRT	10.51	11.66		18.59	21.04		27.25	36.56		51.07	50.53	
14	WB/CW + nodal	3DCRT	6.69	7.19		10.71	11.86		36.99	38.8		50.84	51.43	
15	WB/CW + nodal	3DCRT	5.56	6.06		8.69	9.71		17.06	17.28		48.48	47.27	
16	WB/CW + nodal	IMRT	7.81	6.81		13.88	11.85		36.54	39.63		51.23	51.6	

WB, whole breast, CW, chest wall, WBI, whole breast irradiation, IMRT, intensity-modulated radiotherapy, 3DCRT, 3D conformal radiotherapy

A, Patients treated with 3DCRT + DIBH (n = 88): the difference in heart and LAD doses (DIBH vs. FB/prone) differed according to the case and the dosimetry parameter

B, List of individual dose differences among the 16 patients excluded from DIBH due to unacceptable heart and LAD doses; in these, alternative RT techniques were used as indicate

The relative increase in the volume of the ipsilateral lung was 1.75 ± 0.06 (range 1.25–2.41).

Weak correlations were found in the entire DIBH population between the relative increase of the ipsilateral lung volume and the MHD (R = 0.400, *p* < 0.000), heart V_25Gy_ (R = 0.386, *p* < 0.001) and LAD mean dose (R = 0.242, *p* = 0.037). None of the studied patient-related or RT parameters were associated with the benefit of DIBH.

In a dosimetry analysis, we studied the nodal doses in a cohort of 30 WBI cases under FB *vs.* DIBH. WBI with DIBH delivered significantly less dose to the level 1 axillary lymph nodes but larger doses to the interpectoral and internal mammary nodes than WBI under FB (Table 4). Although the average V_45 Gy_ was < 30% in all subregions, individual dose coverage differed (Table 3).

**Table 3:** Nodal coverage among the WBI cases under FB vs. with DIBH

n = 30	Nodal subregion	FB	DIBH	*p*
Volume (cm^3^)	Axillary Level 1	51.8 ± 28.3	51.9 ± 28.4	0.692
	Subpectoral	11.3 ± 3.7	11.3 ± 4.0	0.824
	Interpectoral	5.0 ± 2.8	5.1 ± 2.8	0.204
	Axillary Level 3	10.5 ± 3.0	10.4 ± 3.3	0.451
	Axillary Level 4	13.6 ± 3.3	13.5 ± 3.2	0.422
	Internal Mammary	1.5 ± 0.4	1.4 ± 0.4	0.004
Mean dose (Gy)	Axillary Level 1	29.5 ± 9.2	26.1 ± 9.4	0.005
	Subpectoral	7.7 ± 5.1	7.8 ± 5.5	0.903
	Interpectoral	26.3 ± 10.2	30.2 ± 9.1	0.024
	Axillary Level 3	3.2 ± 2.3	3.6 ± 3.8	0.538
	Axillary Level 4	0.8 ± 0.2	0.8 ± 0.2	0.209
	Internal Mammary	13.5 ± 8.3	17.3 ± 10.6	0.008
V > 25 Gy (%)	Axillary Level 1	60.2 ± 23.1	51.7 ± 22.6	0.005
	Subpectoral	10.0 ± 11.4	10.7 ± 13.0	0.716
	Interpectoral	55.6 ± 24.2	63.9 ± 22.4	0.036
	Axillary Level 3	2.0 ± 3.7	3.0 ± 7.5	0.422
	Axillary Level 4	0.0	0.0	–
	Internal Mammary	19.8 ± 21.3	29.9 ± 27.4	0.007
V > 45 Gy (%)	Axillary Level 1	31.3 ± 19.6	27.2 ± 20.3	0.169
	Subpectoral	0.5 ± 2.2	0.3 ± 1.0	0.705
	Interpectoral	17.1 ± 18.2	25.2 ± 23.9	0.068
	Axillary Level 3	0.1 ± 0.3	0.1 ± 0.2	0.795
	Axillary Level 4	0.0	0.0	–
	Internal Mammary	6.4 ± 14.9	12.3 ± 20.1	0.065
V > 47.5 Gy (%)	Axillary Level 1	13.2 ± 13.8	13.0 ± 15.1	0.932
	Subpectoral	0.1 ± 0.1	0.0	0.326
	Interpectoral	5.0 ± 13.2	8.3 ± 16.5	0.223
	Axillary Level 3	0.0	0.0	–
	Axillary Level 4	0.0	0.0	–
	Internal Mammary	3.8 ± 11.1	5.8 ± 11.6	0.111

FB, free breathing; DIBH, deep inspirational breath hold

### Feasibility

All the patients having started DIBH completed the treatment. Inter-fractional random and systematic setup errors were 3.9 and 2.7 mm, respectively. The average and median time for daily radiotherapy with DIBH took mean: 10.7 (7–19) minutes.

In 43 cases including 27 patients with WBI only, 4 with WBI + nodal RT and 12 with CW + nodal RT, post-RT follow-up data were analyzed (Table 4). Radiodermatitis of grade 1 was usual breast fibrosis occurred in 40% of the patients 3 months after the RT but was much less thereafter. Asymptomatic inflammatory/fibrotic radiogenic lung changes were present in most cases, while in 2 patients also chest pain and cough developed, but medical intervention was not needed (pneumonitis of grade 1) (Table 4/A). Visual assessment and MLDC data were compared to the same parameters of a cohort of patients having received WBI in the supine position under FB in 2010–2011; no significant difference was detected (Table 4/B). Good agreement between visual scores and MLDC values 3 months after RT was found; no association was detected with the relative change of the ipsilateral lung volume.

**Table 4:** Radiogenic side effects at the end of RT under DIBH and 3 months or 1 year thereafter

Grade	Acute dermatitis (n = 43)	3 months after RT (n = 43)			12 months after RT (n = 35)		
		Pneumonitis	Dermatitis	Breast fibrosis	Pneumonitis	Dermatitis	Breast fibrosis
(A)							
0	3	41	14	26	35	30	29
1	33	2	29	15	0	5	4
2	7	0	0	2	0	0	2

		3 months after RT		p (ANOVA)	12 months after RT		p (ANOVA)
		n (%)	MLDC		n (%)	MLDC	
(B)							
3DCRT + DIBH	visual score 0	6 (14.0)	12.0 ± 38.4	< 0.001	8 (22.9)	37.3 ± 83.1	0.119
	Visual score 1	20 (46.5)	49.4 ± 41.4		15 (42.9)	49.6 ± 30.6	
	Visual score 2	17 (39.5)	126.4 ± 54.9		12 (34.2)	95.7 ± 92.5	
3DCRT + FB	visual score 0	7 (28.0)	32.4 ± 32.6	0.035	6 (40.0)	20.3 ± 53.5	0.522
	Visual score 1	11 (44.0)	43.1 ± 19.3		6 (40.0)	33.5 ± 21.8	
	Visual score 2	7/25 (28.0)	71.4 ± 32.1		3 (20.0)	53.7 ± 37.8	

MLDC: mean lung density changes

A, Toxicity was assessed according to the CTC AE vs. 4 system

B, Lung density changes by means of visual assessment and lung density measurements were compared to the same parameters of a historical cohort of patients having received WBI in the supine position under FB in 2012; no significant difference was found

During the RT of the first 37 patients, both patients and technicians evaluated the feasibility and easiness of the DIBH technique by completing a questionnaire. Patients were asked about their comfort during the DIBH procedure, the stability of the position and the easiness of the intervention, while radiographers were asked about their efforts needed and the compliance of the patient during intervention. High total scores indicate satisfaction with the method from both sides (Table 5).

**Table 5:** Acceptance of the DIBH method by the patient (at the occasions of RT fractions 6 and 25) and the radiographer (at the occasions of planning CT and RT fractions 6 and 25) was scored in 37 cases; the patients answered the same 3 questions, the radiographers the same 4 questions each time. Score 0: worst, score 3: best; scores to specific questions were added up

n = 37	Patient (maximum score: 9)		Radiographer (maximum score: 12)		
	Fraction 6	Fraction 25	Planning CT	Fraction 6	Fraction 25
Mean ± SD	7.9 ± 1.3	7.4 ± 1.5	9.9 ± 2.1	9.2 ± 2.1	9.5 ± 1.8
Median	8	7	10	9	10
Range	5–9	5–9	4–12	5–12	5–12

## Discussion

DIBH could not be implemented for all patients. We found that even among patients seemingly appropriate for the technique, about 20% could not practise DIBH, and 15% of the rest due to dosimetry concerns had to receive RT using an alternative technique. Furthermore, although in the remaining cases the DIBH maneuver resulted in reduced heart and LAD and lung doses in most, still, in some cases FB or prone positioning yielded superior or equal dosimetry results.

In a large database of 272 patients, similar experience was found: more than 40% of the patients were not suitable for or did not benefit from 3DCRT + DIBH [25]. Tanguturi et al. found that among 146 patients deemed potential candidates for breast radiotherapy with DIBH, the DIBH technique provided a neutral change in 25 (17%) or even increased MHD in 14 (10%) cases. A beneficial effect was favored by younger age, greater body mass index (BMI) and larger inspirational lung volume changes [26]. Dell’Oro et al. demonstrated that the benefit provided by DIBH in OAR doses individually differs. Notably, 3 out of 20 patients had higher heart, 4 had increased LAD and 6 had increased lung doses under DIBH as compared to FB [19]. It was found that larger total lung volume increase predisposed to more significant DIBH-related heart and LAD dose reductions. Lin et al. found that in comparison to FB, DIBH resulted larger benefit in heart and LAD doses during postmastectomy RT than during WBI [27]. No similar relationship could be seen in our study; in this patient cohort heart and LAD dose reductions were weakly correlated with DIBH-related lung volume increases, and none of the studied parameters seemed to have predictive power.

Due to the mentioned obstacles of benefiting the most from DIBH and for the optimal use of resources, every RT center should decide its strategy how to include DIBH into routine practice. One approach is the use of anatomical attributes for individual patient selection. Lin et al. tested artificial intelligence-based predictive models to choose the preferred RT technique DIBH *vs.* prone in a set of 16 patients needing WBI. Taking into consideration heart doses only, breast volume and the distance between the center of the breast and the heart, while respecting composite OAR exposure including the ipsilateral lung, the volumes of the heart and breast, and breast-lung distance had predictive power [27]. If just heart doses were considered, in more cases was preferred the DIBH technique, while if weighted OAR toxicity (dose to heart, ipsilateral lung, and contralateral breast) was considered, the opposite was the finding.

Another possibility is to apply DIBH only in a selected group of patients for example if heart exposure seems unfavorable under FB. Such an optimization-selection approach was the development of a trained model (using RapidPlan™, Varian) to develop RT preplans for the estimation of heart doses under FB: if the parameters under FB are satisfying, there is no need for a second series of CT under DIBH [28]. Another strategy to perform screening of all left-sided cases for DIBH using a quick, pragmatic, and systematic assessment protocol by radiographers to consider whether the technique is beneficial, and the patient is suitable [25]. In both cases, primary CT scanning under FB was needed. Tanna et al. compared 4 selection methods, and found optimal the London Cancer Alliance’s upfront selection process (without the need of CT scanning under FB) that recommends the use of DIBH if the tumor bed is situated in the lower quadrants of the breast or is extensive, and in all CW RT cases [29]. This method should be preferred in centers with resource constraints.

In most cases similar heart and LAD dose reductions to published results were found at DIBH [17, 18, 20]. We consider DIBH one of the heart sparing techniques which, has the mainstay of good reproducibility and the position-stabilization of the PTV [30]. In WBI cases an alternative solution could be prone positioning; in this, although repositioning accuracy is inferior, ipsilateral lung dose is significantly reduced. In nodal RT cases, if DIBH cannot be utilized, the IMRT technique may provide solution. Actually, Zhao et al. found that unacceptable heart and LAD doses in 3DCRT plans with DIBH could be solved using the IMRT technique, but no further dosimetric improvement was seen in low OAR-dose cases [31]. In fact, the consideration of the dose constraints of the heart and its substructures seems the most important for adequate heart sparing in modern RT as suggested by the DEGRO breast cancer expert panel; the appropriate technique should be selected accordingly [32].

Thanks to the successful use of new technologies and raised attention to solving heart sparing, the control of ipsilateral lung doses is becoming a challenge. It has been estimated that DIBH by reducing the dose to the ipsilateral lung volume and lung mass by 15–24%, results in an about 20% improvement in lung normal tissue complication probability (NTCP) as compared to that after FB [33, 34]. In fact, lung doses approximate zero if prone WBI is performed. Yan et al. based on literature data, compared the risks of cardiac death, significant cardiac event, or lung cancer mortality in 34 breast cancer patients having RT plans both under DIBH and in the prone position. Both techniques provided excellent cardiac sparing, nevertheless, with DIBH, an excess lung cancer mortality of 0.5% evolved, which was absent with the prone WBI technique [35]. We studied the occurrence of acute and late radiation changes of the ipsilateral lung by means of visual evaluation and lung density measurements. That special interest was due to the known stimulatory effect of oxygen on radiosensitivity. The incidence of asymptomatic abnormalities was higher, but not significantly different from that in an earlier patient cohort (Table 4/B). We believe that the protocol-defined follow-up period of 1 year post-radiotherapy was sufficient to detect all adverse pulmonary reactions.

A significant proportion of the enrolled patients was not able to effectively practise DIBH. Those whose voluntary breath-holding capacity or chest wall movements were not sufficient for DIBH were identified before or at simulation. In a retrospective analysis, Nissen et al. found similar proportion and similar reasons for omitting DIBH in a left-sided BC patient population: among 20 patients non-compliant to DIBH, 7 could not use the equipment, 7 was not able to maintain breath-hold for at least 20 s, 7 had psychological problems, and 4 had other reasons [36]. Training for DIBH is essential. Kim et al. detected lower heart doses in patients who received 5-day preparatory coaching before starting DIBH versus those who did not [36]. There is controversy whether thoracic or abdominal DIBH provides better heart sparing [31]. Zhao et al. found that most patients practice thoracic DIBH, but may be easily trained for abdominal DIBH which in fact, better lowers heart, LAD and lung doses [31]. Proper DIBH technique is essential since its beneficial effect depends primarily on the lung volume increase achieved [19, 26]. We consider very important the role of the physiotherapist expert who should provide standardized coaching and support to the patients. The DIBH technique was easily adapted, and inter-fractional repositioning accuracy seemed similar to that of other techniques (supine, prone, PBI).

As demonstrated by Pazos et al. and Borm et al. [37, 38], during DIBH, the relative position of the nodal regions to that of the PTV changes and as a consequence accidental doses to axillary levels I and II become less or more relevant as compared to that without DIBH. We separately analyzed the 6 nodal regions, and found that due to DIBH while the dose to axillary level I is lower, the doses to the interpectoral lymph node region and the ipsilateral internal mammary (IM) region may be higher than during FB, however, differences show individual variations. We think that this finding should raise attention to always considering the doses to axillary levels I-II if their irradiation is needed due to limited axillary surgery and low-volume SNB positivity. In these cases, supine RT with DIBH should be favored instead of prone WBI since during the latter the axillary lymph node regions are excluded from irradiation [23].

Since the dose to the OARs is rarely a problem during PBI, limited experience is published with PBI and DIBH [37]. The DIBH method utilized independently of the laterality of the disease was found useful for the PBI of 37 patients [39]. In our practice, heart and LAD exposure clearly depended on the size and location of the tumor bed. Recently, the use of DIBH occasionally, in cases with relatively high heart or LAD doses in PBI plans the DIBH method provided prompt solution.

## Conclusions

DIBH is one of the powerful heart sparing techniques in breast cancer RT. Alertness is needed to identify those patients who do not benefit from that laborious procedure or benefit less than from an alternative method: if WBI is needed, prone positioning, if nodal RT is necessary the IMRT technique could be alternate options.

## Supplementary information

**Additional file 1**.

## Data Availability

The datasets used and/or analysed during the current study are available from the corresponding author on reasonable request.

## Abbreviations

3DCRT: 3-Dimensional conformal radiotherapy
CW: Chest wall
DIBH: Deep-inspirational breath-hold
ESTRO: European Society for Radiotherapy and Oncology
FB: Free breathing
LAD: Left anterior descending coronary artery
MHD: Mean heart dose
OAR: Organ at risk
PBI: Partial breast irradiation
PMI: Postmastectomy irradiation
PTV: Planning target volume
RIHD: Radiation-induced heart disease
RT: Radiotherapy
SNB: Sentinel node biopsy
V_xGy_: A subvolume of a volume that is exposed to ≥ x Gy
WBI: Whole breast irradiation
