# Supplementary material for: Deep-inspirational breath-hold (DIBH) technique in left-sided breast cancer: various aspects of clinical utility
Source: Radiat Oncol. 2021 May 13;16:89. doi: 10.1186/s13014-021-01816-3 (PMC8117634; doi:10.1186/s13014-021-01816-3)
Supplement: Supplementary file 1 — Additional file 1. [file 13014_2021_1816_MOESM1_ESM.docx]

Supplementary Table S1 Questionnaires for the patients to be filled out after RT (fractions 6 and 25) and the personnel at the planning CT and RT (fractions 6 and 25); high scores indicated satisfactory acceptance

| *Patients* | *Radiographers* |
| --- | --- |
| **Please answer the following questions by agreeing as:**  0: not at all, 1: a little, 2: moderately, 3: very much  How comfortable did you feel during the treatment?  0 1 2 3  How did you succeed in keeping the treatment position?  0 1 2 3  How easy was it to go through the treatment? 0 1 2 3 | **Please answer the following questions by agreeing as:**  0: not at all, 1: a little, 2: moderately, 3: very much  Was it easy to position the patient? 0 1 2 3  How cooperative was the patient during positioning?  0 1 2 3  How comfortable was the procedure for the patient?  0 1 2 3  How simple was it to cross-check the superficial and laser signs?  0 1 2 3 |

.
